# Solr-Plant: efficient extraction of plant names from text

**DOI:** 10.1186/s12859-019-2874-6

**Published:** 2019-05-22

**Authors:** Vivekanand Sharma, Maria Isabel Restrepo, Indra Neil Sarkar

**Affiliations:** Center for Biomedical Informatics, Brown University, Box G-R, Providence, RI USA

**Keywords:** Biodiversity informatics, Taxonomic name recognition, Plant name identification

## Abstract

**Background:**

The retrieval of plant-related information is a challenging task due to variations in species name mentions as well as spelling or typographical errors across data sources. Scalable solutions are needed for identifying plant name mentions from text and resolving them to accepted taxonomic names.

**Results:**

An Apache Solr-based fuzzy matching system enhanced with the Smith-Waterman alignment algorithm (“Solr-Plant”) was developed for mapping and resolution to a plant name and synonym thesaurus. Evaluation of Solr-Plant suggests promising results in terms of both accuracy and processing efficiency on misspelled species names from two benchmark datasets: (1) SALVIAS and (2) National Center for Biotechnology Information (NCBI) Taxonomy. Additional evaluation using S800 text corpus also reflects high precision and recall. The latest version of the source code is available at https://github.com/bcbi/SolrPlantAPI. A REST-compliant web interface and service for Solr-Plant is hosted at http://bcbi.brown.edu/solrplant.

**Conclusion:**

Automated techniques are needed for efficient and accurate identification of knowledge linked with biological scientific names. Solr-Plant complements the current state-of-the-art in terms of both efficiency and accuracy in identification of names restricted at species level. The approach can be extended to identify broader groups of organisms at different taxonomic levels. The results reflect potential utility of Solr-Plant as a data mining tool for extracting and correcting plant species names.

## Background

Plant-related information is embedded across biodiversity and biomedical data sources. Acquisition of relevant information from such heterogeneous sources is a challenging task. Unlike ongoing efforts in the biomedical community to make data publicly accessible in a standardized form, there are fewer tools and techniques for biodiversity text mining. The diverse nature of species name mentions including the presence of ambiguous, synonymous, or misspelled terms poses a bottleneck in the standardization of available data [5]. A requirement for such tasks is the ability to resolve names used in data sources to accepted taxonomic names. This is an essential step towards supporting the linking of knowledge across biodiversity data sources, acknowledging the species-centric nature of the discipline [18]. This reconciliation must also include mapping of name variants to taxonomic concepts.

Misspellings, inconsistencies in author abbreviations, and mentions of non-accepted synonyms may result in information loss. The issues and impact of ambiguous and erroneous mentions of botanical names in peer-reviewed literature have been discussed by Rivera et al. [15]. Misspellings or typographical errors associated with organism names in biomedical or biodiversity repositories may affect retrieval of essential information. For example, spelling errors are seen in systems for tracking adverse health events (e.g., herb names) [17]. These challenges require ‘taxonomically intelligent’ strategies to organize relevant information into reconciliation groups [18]. Inability to overcome such issues may limit research that crosses the domains of biodiversity and biomedicine, such as identifying medicinal applications of plants [19]. Lack of species name standardization may further accentuate the risk of erroneous scientific conclusions [3]. There have been efforts in addressing the challenge of taxonomic name resolution. However, correcting misspelled names remains an issue (e.g., in Tropicos [*http://services.tropicos.org*]) and Catalogue of Life (COL) [16]. Spelling correction using fuzzy matching has been implemented in resources like Plantminer [7], Global Names Resolver (GNResolver: *http://resolver.globalnames.org/*), Taxamatch [14], and Taxonomic Name Resolution Service (TNRS) [5]. TNRS uses the fuzzy matching capabilities of Taxamatch and has shown improved performance when compared to Plantminer and GNResolver. Here, we describe “Solr-Plant” as a tool that utilizes taxonomic names from a combination of three validated sources in conjunction with fuzzy search capabilities that harness the Apache Solr [1]) and Smith-Waterman string alignment [20] algorithms. The results demonstrate the utility of Solr-Plant to support mining of plant species names from natural language text.

### Implementation

The goal of this study was to build a tool for identification and normalization of plant species names. A collection of organism names (uBiota) was compiled by unification of taxonomy from three different sources: (1) Catalogue of Life (COL) [16]; (2) Integrated Taxonomic Information System (ITIS) (https://www.itis.gov/); and (3) National Center for Biotechnology Information (NCBI) Taxonomy [8]. uBiota unique identifiers were assigned to taxonomic units representing canonical Linnaean taxonomic groups: Kingdom, Phylum, Class, Order, Family, Genus, and Species. This study focused on taxonomic units belonging to kingdom Plantae. Synonyms for accepted plant species names were then gathered from the source databases.

The compendium of plant names was indexed using Apache Solr (7.0.1), an open source search platform. A search query was then generated to leverage Solr’s fuzzy matching capabilities using the input name string to retrieve relevant matches from the indexed uBiota dictionary. The top ten records in order of match score were considered for further processing. The next step consisted of calculating local alignments between the query string and ten best-retrieved records using the Smith-Waterman algorithm. The top scoring alignment region was retained after considering the following constraints: (1) The coverage of aligned string to retrieved record was greater than 80%; and (2) The first characters of the genus and species epithet matched. The resulting uBiota record with maximum alignment score was assigned a binary decision as ‘match’ or ‘no-match.’

The algorithmic implementation of the system (“Solr-Plant”) was done using Julia (v.1.0) [2]. Solr-Plant is accessible as a Representational State Transfer (REST)-compliant web service (http://bcbi.brown.edu/solrplant_api/?plantname=<input string>). The source code is available on Github at https://github.com/bcbi/SolrPlantAPI. Solr-Plant was evaluated by comparison to other systems on two datasets: (1) 1000 uncorrected plant names from the SALVIAS [6] project [4]; and (2) Misspelled plant species names provided as part of NCBI Taxonomy [12]. The parameters used for all the systems were set to retrieve best matches using their respective API calls. The default parameter for TNRS uses all the sources of taxonomic names. These sources include The Plant List (TPL) [21]), Global Composite Checklist (GCC) [9], International Legume Database and Information Service (ILDIS) [11], TROPICOS [22], and United States Department of Agriculture (USDA) [23]. TPL was used as the source for fuzzy matching taxonomic names using Plantminer. Application Programming Interface (API) calls to GNResolver were made for resolving names by fuzzy match criteria against sources restricted to NCBI, COL, and ITIS.

Additional evaluation was performed using article abstracts from the S800 corpus [13]. This corpus consists of annotated abstracts from eight categories: bacteriology, botany, entomology, medicine, mycology, protistology, virology, and zoology. For the purpose of this study, abstracts belonging to the botany category (S800: Botany) were selected. The pre-processing of article abstracts consisted of two additional steps: (1) Sentence tokenization; and (2) Noun phrase detection. Precision and recall metrics were used to evaluate the performance on text dataset with a focus on detecting plant names at the species level (*Genus species*).

## Results

Solr-Plant processed 1000 names in 20.00 s locally (on an i7 3.5 GHz machine with 16 GB RAM running MacOS 10.13.5) and in 67.25 s using the web API as compared to 359.88, 286.19, and 262.21 s for TNRS, Plantminer, and GNResolver, respectively, when names were processed individually. Previously reported speed benchmark results for batch processing from TNRS, Plantminer, and GNResolver were 43 s, 613 s, and 312 s respectively [5]. A comparative report of the plant name mappings on two evaluation datasets are provided in Tables 1 and 2. Out of the 36 identified as false matches by Solr-Plant on the SALVIAS dataset, six were incorrect and the remaining were partial matches (covering only the genus names). The performance of Solr-Plant was slightly lower compared to TNRS (F-score: 0.95 versus 0.98); however, the approach itself showed better performance on normalizing misspelled names as shown on NCBI misspelling dataset. The processing speed of the respective web APIs was also evaluated, with Solr-Plant having better performance (Table 2). The time taken by Solr-Plant to process 6411 records from NCBI misspelling dataset locally was 105 s.

**Table 1:** Performance comparison on SALVIAS dataset

	Solr-Plant	TNRS^a^	Plantminer^a^	GNResolver^a^
TRUE	950	980	881	745
FALSE	36	19	43	29
NOT FOUND	14	1	76	226
TOTAL	1000	1000	1000	1000
PRECISION	0.96	0.98	0.95	0.96
RECALL	0.95	0.98	0.88	0.74
F-SCORE	0.95	0.98	0.91	0.84
TIME (Batch)	NA	43 s	613 s	72 s
TIME^b^ (Individual)	67.25 s	359.88 s	286.19 s	262.21 s

^a^Evaluation results as provided by Boyle *et al.*, 2013; ^b^ Time comparison conducted for this study

**Table 2:** Performance comparison on NCBI misspelling dataset

	Solr-Plant	TNRS	Plantminer	GNResolver^a^
TRUE	5723	5302	4333	3761
FALSE	625	995	685	2581
NOT FOUND	63	114	1393	69
TOTAL	6411	6411	6411	6411
PRECISION	0.90	0.84	0.86	0.59
RECALL	0.89	0.83	0.68	0.59
F-SCORE	0.89	0.83	0.67	0.59
TIME	362.36 s	2084.86 s	1710.19 s	2039.56 s

^a^For GNResolver the ‘best match’ criterion was used for mappings

Evaluation using the text corpus dataset (S800: Botany) based on the criteria of identification of plant names at the species level (binomial nomenclature) resulted in a precision of 0.9765 and recall of 0.9765. The collection of 100 abstracts were processed in 38.64 s.

## Discussion

Mobilizing and linking organism indexed data within and across biodiversity and biomedical domains commonly rely upon species-centric approaches. The prerequisites for such an approach include the correct identification and reliable normalization of taxonomic entities to accepted names in a scalable manner. However, the execution of species name-based analytic techniques for high-throughput data extraction spanning repositories is fraught with the challenges of name variants and erroneous names due to spelling or typographical errors. As demonstrated here, a Solr-based approach is able to resolve plant names in a highly accurate and efficient manner (Table 1). The performance is slightly limited by the taxonomic name coverage (e.g., compared to systems like TNRS). However, considering the task of resolving misspelled names, Solr-Plant performs better in terms of: (1) correctness of mappings; and (2) processing time (Table 2). This performance may be attributed to efficient indexing and search capabilities within the Apache Solr framework in comparison with matching algorithms previously used for plant name identification. Additional considerations related to non-uniformity of source databases should also be taken into consideration while interpreting results (sources listed in the Implementation section). The differences in number of unmatched plant species names may arise as a result of their absence from source databases. For example, from the evaluation of TNRS (Table 2) 65 out of 114 unmatched plant species were not present in source databases. Although non-uniformity of source databases is an issue, the total number including incorrect matches as well as those present and unmatched is much higher for TNRS.

The candidate name strings are distilled in a final step using the Smith-Waterman algorithm. The advantage of using string alignment over other metrics used to measure string distances is that it allows for the best matching query substring with allowable edit operations (insertion, deletion, or substitution). To further characterize the utility of the approach developed in this study, performance was evaluated on S800: Botany corpus. The precision and recall values were high (0.9765 and 0.9765 respectively, F-score 0.9765) when considered for identification of binomial names currently restricted at species level. However, the precision value was low (0.58) when single word mappings were included. Such an issue could be addressed by additional processing steps such as use of a negative word lexicon. A comparative evaluation of the system previously implemented by Pafilis et al. (SPECIES [13]) with LINNAEUS [10] on S800: Botany corpus resulted in an F-score of approx. 0.8746 and 0.8924 respectively. Both these systems use common list of words to avoid false positives. The current version of Solr-Plant does not match authorities. Future work will aim at extending to include matching of authorities and distinguishing between homonyms. Given the flexibility of Apache Solr and mapping based on Smith-Waterman those are achievable goals. However, addition of such functionality will require having a more comprehensive dictionary containing valid representation of authority and links to single best accepted name. The results indicate that while accommodating for misspelled species names, the precision of Solr-Plant is not compromised, which may be a reason of concern for approximate matching approaches. This study highlights the potential of Solr-Plant as a text mining tool for extraction and correction of plant species names. Such features may be used to support processing of text derived from optical character recognition (OCR). Additional possible enhancements of the Solr-Plant tool include distributed indexing and load-balanced querying capabilities for full-text search and high volume processing. Future versions may also include expansion to broader taxonomic name recognition beyond plant species.

## Conclusions

The effective extraction and resolution of taxonomic names in a scalable manner represents an important aspect of informatics-based applications for organizing and studying plant-related information. Solr-Plant is a complementary tool to the current state-of-the-art plant species taxonomic name recognition in terms of both efficiency and accuracy. The approach may be extended for identifying broader groups of organisms. The results reflect the feasibility of using this tool for efficiently extracting and correcting plant species names from text with misspellings.

## Abbreviations

API: Application Programming Interface
COL: Catalogue of Life
GCC: Global Compositae Checklist
GNResolver: Global Names Resolver
ILDIS: International Legume Database and Information Service
ITIS: Integrated Taxonomic Information System
NCBI: National Center for Biotechnology Information
OCR: Optical Character Recognition
REST: Representational State Transfer
TNRS: Taxonomic Name Resolution Service
TPL: The Plant List
uBiota: Collection of organism names
USDA: United States Department of Agriculture

## References

[1] “Apache Solr.” 2011. http://lucene.apache.org/solr/.

[2] Bezanson J et al. 2012. “Julia.” 2012. https://julialang.org/.

[3] Bortolus A (2008). Error Cascades in the Biological Sciences: The Unwanted Consequences of Using Bad Taxonomy in Ecology. Ambio.

[4] Boyle, Brad, Nicole Hopkins, Zhenyuan Lu, Juan Antonio Raygoza Garay, Dmitry Mozzherin, Tony Rees, Naim Matasci, et al. 2013a. “1000 Uncorrected Plant Names from SALVIAS.” 2013. https://static-content.springer.com/esm/art%3A10.1186%2F1471-2105-14-16/MediaObjects/12859_2012_5617_MOESM2_ESM.csv. 10.1186/1471-2105-14-16 PMC3554605 23324024

[5] Boyle, Brad, Nicole Hopkins, Zhenyuan Lu, Juan Antonio Raygoza Garay, Dmitry Mozzherin, Tony Rees, Naim Matasci, et al. 2013b. “The Taxonomic Name Resolution Service: An Online Tool for Automated Standardization of Plant Names.” *BMC Bioinformatics* 14 (January): 16. 10.1186/1471-2105-14-16 PMC3554605 23324024

[6] Boyle Bradley, Enquist Brian (2012). SALVIAS – the SALVIAS vegetation inventory database. Biodiversity & Ecology.

[7] Carvalho GH, Cianciaruso MV, Batalha MA (2010). Plantminer: A Web Tool for Checking and Gathering Plant Species Taxonomic Information. Environ Model Softw.

[8] Federhen S. (2011). The NCBI Taxonomy database. Nucleic Acids Research.

[9] gbif.org, Registry-Migration. 2015. “Global Compositae Checklist (GCC).” International Compositae Alliance. 10.15468/G7YHGT.

[10] Gerner Martin, Nenadic Goran, Bergman Casey M (2010). LINNAEUS: A species name identification system for biomedical literature. BMC Bioinformatics.

[11] “ILDIS.” 2018. International Legume Database and Information Service. 2018. https://www.ildis.org/.

[12] NCBI. 2011. “NCBI Taxonomy Dataset Download.” 2011. https://ftp.ncbi.nlm.nih.gov/pub/taxonomy/new_taxdump/.

[13] Pafilis E, Frankild SP, Fanini L, Faulwetter S, Pavloudi C, Vasileiadou A, Arvanitidis C, Jensen LJ (2013). The SPECIES and ORGANISMS Resources for Fast and Accurate Identification of Taxonomic Names in Text. PLoS One.

[14] Rees T (2014). Taxamatch, an Algorithm for near (‘fuzzy’) Matching of Scientific Names in Taxonomic Databases. PLoS One.

[15] Rivera D, Allkin R, Obón C, Alcaraz F, Verpoorte R, Heinrich M (2014). What Is in a Name? The Need for Accurate Scientific Nomenclature for Plants. J Ethnopharmacol.

[16] Ruggiero M, Gordon D, Bailly N, Kirk P, Nicolson D, Bisby FA, Roskov YR (2009). “The Catalogue of Life Taxonomic Classification.” Edition.

[17] Sakaeda T, Tamon A, Kadoyama K, Okuno Y (2013). Data Mining of the Public Version of the FDA Adverse Event Reporting System. Int J Med Sci.

[18] Sarkar IN (2007). Biodiversity Informatics: Organizing and Linking Information across the Spectrum of Life. Brief Bioinform.

[19] Sharma V, Sarkar IN (2013). Leveraging Biodiversity Knowledge for Potential Phyto-Therapeutic Applications. Journal of the American Medical Informatics Association: JAMIA.

[20] Smith TF, Waterman MS (1981). Identification of Common Molecular Subsequences. J Mol Biol.

[21] “TPL.” 2013. The Plant List. 2013. http://www.theplantlist.org/.

[22] “Tropicos.” 2018. 2018. https://www.tropicos.org/.

[23] “USDA, NRCS.” 2018. The PLANTS Database. 2018. http://plants.usda.gov.

